# Pre-treatment inflammatory parameters predict survival from endometrial cancer: A prospective database analysis

**DOI:** 10.1016/j.ygyno.2021.11.009

**Published:** 2022-01

**Authors:** Kelechi Njoku, Neal C. Ramchander, Y. Louise Wan, Chloe E. Barr, Emma J. Crosbie

**Affiliations:** a Division of Cancer Sciences, University of Manchester, School of Medical Sciences, Faculty of Biology, Medicine and Health, 5th Floor Research, St Mary's Hospital, Oxford Road, Manchester M13 9WL, United Kingdom; b Stoller Biomarker Discovery Centre, Institute of Cancer Sciences, Faculty of Biology, Medicine and Health, University of Manchester, Manchester, United Kingdom; c Department of Obstetrics and Gynaecology, Manchester University NHS Foundation Trust, Manchester Academic Health Science Centre, Manchester, United Kingdom

**Keywords:** Endometrial cancer, Inflammation, Survival, Prognosis, C-reactive protein, Glasgow prognostic score

## Abstract

**Purpose:**

Inflammation predisposes to tumorigenesis by damaging DNA, stimulating angiogenesis and potentiating pro-proliferative and anti-apoptotic processes. The aim of this study was to investigate whether pre-treatment biomarkers of systemic inflammation are associated with survival outcomes in endometrial cancer.

**Patients and methods:**

Women with endometrial cancer were recruited to a prospective database study. Pre-treatment systemic markers of inflammation, including C-reactive protein (CRP), Glasgow Prognostic Score and lymphocyte-based ratios [neutrophil-lymphocyte ratio (NMR), monocyte-lymphocyte ratio (MLR), systemic immune-inflammation index (SII)], were analysed in relation to overall, endometrial cancer-specific and recurrence-free survival using Kaplan-Meier estimation and multivariable Cox regression.

**Results:**

In total, 522 women of mostly White British ethnicity, with a median age of 66 years (interquartile range (IQR), 56, 73) and BMI of 32 kg/m^2^ (IQR 26, 39) were included in the analysis. Most had low-grade (67.2%), early-stage (85.4% stage I/II), endometrioid (74.5%) tumors. Women with pre-treatment CRP ≥5.5 mg/L had a 68% increase in overall (adjusted HR = 1.68, 95% CI 1.00–2.81, *p* = 0.049) and a two-fold higher cancer-specific mortality risk than those with CRP <5.5 mg/L (adjusted HR = 2.04, 95%CI 1.03–4.02, *p* = 0.04). Absolute lymphocyte count, NLR, MLR and SII were associated with adverse clinico-pathologic factors, but not overall, cancer-specific or recurrence-free survival in the multivariable analysis.

**Conclusion:**

If confirmed in an independent cohort, CRP may offer a simple, low-cost test to refine pre-treatment risk assessment and guide personalised care in endometrial cancer. Our participants were mostly of White British ethnicity and further studies are needed to confirm the utility of CRP as a prognostic biomarker in other populations.

## Highlights

• Pre-treatment markers of inflammation were analysed in relation to endometrial cancer survival in a prospective study.
• Women with elevated CRP of ≥5.5 mg/L had a 68% increase in overall and a two-fold increase in cancer-specific mortality.
• There was no evidence of an effect of pre-treatment CRP on recurrence-free survival.
• Lymphocyte-based scores were associated with adverse clinico-pathologic factors, but not survival outcomes.
• CRP offers a simple, low-cost endometrial cancer prognostic test with potential to refine pre-treatment risk assessment.

## 1 Introduction

Endometrial cancer is the sixth most common malignancy in women worldwide, with an estimated 417,000 cases reported globally in 2020 [1]. In the United Kingdom (UK), it is the fourth most common and the 7th leading cause of death from cancer in women [2]. Over the last decade, endometrial cancer mortality rates have risen by 25% in the UK, with similar trends reported in other high income countries [2]. Whilst most women with endometrial cancer are diagnosed early when curative treatment is likely, a significant minority present with advanced or metastatic disease that heralds a poor prognosis [3].

Accurate endometrial cancer risk assessment is fundamental to ensuring women receive appropriate evidence-based care [4]. Currently, clinico-pathological risk assessment is based on tumor parameters, including International Federation of Gynaecology and Obstetrics (FIGO) surgical stage, tumor grade and histology, lymphovascular space invasion and depth of myometrial invasion [5,6]. The molecular classification of endometrial cancer holds great promise for improving risk stratification beyond these standard clinico-pathological features [7]. Management algorithms also take age, body mass index (BMI) and comorbid status into consideration [3]. However, there is emerging evidence that prognosis is influenced by factors other than traditional clinico-pathological parameters, and that these may help to refine endometrial cancer risk assessment [4,[8], [9], [10]].

Chronic low-grade inflammation is one of the biological mechanisms underpinning endometrial carcinogenesis [11]. Adipose tissue expansion and localized hypoxia accompanying excess body fat creates a chronic pro-inflammatory cytokine milieu of interferons, interleukins and C-reactive protein (CRP) [12,13]. The resulting inflammatory state promotes cellular proliferation and reduces apoptosis, contributing to malignant transformation, tumor growth and progression [14]. Whilst inflammation has been shown to increase risk [15], few studies have explored the potential prognostic utility of systemic inflammatory markers in endometrial cancer [[16], [17], [18], [19], [20], [21], [22], [23], [24]].

CRP is an acute-phase inflammatory protein that correlates with poor outcomes in several adult solid tumors [25,26]. A systematic review found CRP was prognostic in 90% of the 271 included studies [27], but few assessed its utility in the context of endometrial cancer [16,18,28]. Those that did failed to account for important clinical and other prognostic parameters that may explain or confound the association between CRP and survival. The systemic immune inflammation index (SII), a composite score that integrates lymphocyte, platelet and neutrophil counts, is a promising prognostic biomarker in several malignancies, including those of the ovary, breast [29], and endometrium [23,24,30] but there is limited evidence to enable its translation into routine clinical practice. Other inflammation-based parameters with potential prognostic utility that need external validation prior to their clinical use include neutrophil-to-lymphocyte ratio (NLR) and monocyte-to-lymphocyte ratio (MLR) [21].

The aim of this study was to investigate whether pre-treatment biomarkers of systemic inflammation are associated with survival outcomes using a large prospective database of endometrial cancer patients.

## 2 Methods

### 2.1 Study population

Women with endometrial cancer treated between 2010 and 2015 at St Mary's Hospital, a regional specialist centre for the management of gynecological cancers, were eligible for inclusion. All participants consented for their pseudo-anonymized data to be used for future research. Relevant sociodemographic and clinico-pathological data, including age, socioeconomic quintile, BMI, comorbidities, histological subtype, tumor grade and stage, depth of myometrial invasion and lymphovascular space invasion (LVSI) were recorded. We categorized age as <65 and ≥ 65 years, in line with age groupings used in many studies, and women were classified as underweight (BMI < 18.5 kg/m^2^), normal weight (BMI 18.5–24.9 kg/m^2^), overweight (BMI 25–29.9 kg/m^2^) or obese (BMI ≥ 30 kg/m^2^). We classified endometrial cancers according to histological subtype (endometrioid, serous, clear cell, carcinosarcoma) based on expert pathology review by two specialist gynecological pathologists, using FIGO 2009 surgical staging criteria [31].

Most women were treated with total hysterectomy and bilateral salpingo-oophorectomy +/−adjuvant therapy, in line with national and international guidelines [3,6]. Women with grade 1 stage 1a endometrial cancer who wished to preserve their fertility, or who were medically unfit for surgery, received primary hormone therapy (+/−delayed hysterectomy). A few women received primary palliative radiotherapy. We reviewed all cases in follow-up clinics at 3-month (for 3 years), 6-month (for 1 year) and 12-month intervals for a total duration of 5 years, or until disease recurrence or death, whichever was sooner. We contacted GPs to ascertain current status where women had completed routine hospital-based follow up or moved away from Manchester. Disease recurrence was managed according to national and international recommendations [3,5]. Women with pelvic recurrence were managed surgically or with radiotherapy as appropriate, whereas those with metastatic or distant recurrent disease were managed with palliative hormone therapy, chemotherapy +/− radiotherapy [3,6]. We obtained cause of death information from death certificates.

### 2.2 Systemic inflammatory indices

We measured pre-treatment complete blood count (CBC), CRP and albumin levels for the study participants. Glasgow prognostic score (GPS) was calculated as follows: women with CRP > 10 mg/L and albumin<35 g/L were allocated GPS = 2; those with CRP > 10 mg/L or albumin<35 g/L were allocated GPS = 1; and those with CRP ≤ 10 mg/L and albumin≥35 g/L were allocated GPS = 0. Modified GPS (mGPS) was calculated as follows: women with CRP > 10 mg/L and albumin<35 g/L were allocated mGPS = 2; women with CRP > 10 mg/L and albumin≥35 g/L were allocated mGPS = 1; and those with CRP < 10 mg/L were allocated mGPS = 0. The following lymphocyte-based ratios were calculated: Neutrophil to Lymphocyte Ratio (NLR: neutrophil divided by lymphocyte count), Monocyte to Lymphocyte Ratio (MLR: monocyte divided by lymphocyte count), and Systemic Immune Inflammation Index (SII: neutrophil multiplied by platelet and divided by lymphocyte count. For each biomarker, the most appropriate cut-off value was based on the optimal decision threshold derived from receiver operating characteristics (ROC) curve analysis. The main study endpoints were overall, cancer-specific and recurrence-free survival.

### 2.3 Statistical analysis

Overall survival was defined as the time interval in months from start of primary treatment to death from any cause or the last day of survival data available. Cancer-specific survival was calculated from start of primary treatment to death from endometrial cancer or the date of last follow-up and censored on date of death from other causes. Recurrence-free survival was calculated from start of primary treatment to first record of recurrence, death or date of last follow-up, whichever was sooner. All inflammatory markers were analysed as both continuous and categorical variables (based on ROC defined thresholds). Chi-square (X^2^) and Fisher's exact tests were used to compare proportions between groups, as appropriate. Student's *t*-test and one-way or two-way ANOVA was used to test for statistical significance as indicated. The Kaplan–Meier method was used to compute survival rates and the log-rank test assessed survival differences between groups. Cox regression multivariable modeling was used to measure the association between inflammatory parameters and survival after adjustment for confounding and effect modifications. Hazard ratios (HRs) with 95% confidence intervals (95% CIs) were computed for both univariable and multivariable analyses. The confounding variables included in the models were age at diagnosis, BMI, type 2 diabetes mellitus (T2DM) status, treatment modality, FIGO stage, histological subtype, grade, LVSI, and depth of myometrial invasion. Confounding was evaluated by assessing changes in hazard coefficients following the introduction of these variables to the Cox regression models. The assumption of proportional hazards was assessed and met for all models. A *p*-value of <0.05 was considered statistically significant. All analyses were conducted using the statistical package Stata 16.0 (https://www.stata.com).

## 3 Results

### 3.1 Study population

Of 537 eligible women, pre-treatment lymphocyte-based and CRP levels were available for 467 and 358 women, respectively (Table 1). Their median age and BMI were 66 years (Interquartile range (IQR), 56, 73) and 32 kg/m2 (IQR 26, 39) respectively. Most had low-grade (67.2%), early-stage (85.4% stage I/II), endometrioid (74.5%) cancers. Most women were managed with primary surgery (88.5%), of whom 45% received adjuvant therapy. During the study period, 76 women (14.6%) relapsed, 108 (20.7%) died, and the remainder were alive as of 31 March 2021.

**Table 1:** Socio-demographic characteristics of the study population.

Variable	n (% total)
Age at diagnosis	Median age 66 years (IQR 56, 73)
<65 years	237 (45.4%)
≥65 years	285 (54.6%)
Body Mass Index (kg/m^2^)	Median BMI 32 kg/m^2^ (IQR 26, 39)
Underweight	6 (1.2%)
Normal weight	82 (15.7%)
Overweight	125 (24.0%)
Obese	309 (59.2%)
Tumor grade	
1	245 (45.0%)
2	116 (22.2%)
3	171 (32.8%)
Tumor stage	
I	391 (75.1%)
II	54 (10.4%)
III	69 (13.2%)
IV	7 (1.3%)
Histology	
Endometrioid	389 (74.5%)
Non-endometrioid	133 (25.5%)
Lymphovascular invasion(*n* = 536)	
No	371 (71.5%)
Yes	148 (28.5%)
Depth of myometrial invasion	
<50%	335 (64.2%)
≥50%	187 (35.8%)
Social deprivation group (*n* = 532)	
Social group I (Least deprived)	147 (28.2%)
Social group II (Middle group)	181 (34.7%)
Social group III (Most deprived)	194 (37.2%)
History of type 2 diabetes mellitus (*n* = 535)	
Yes	105 (20.2%)
No	414 (79.8%)
Primary treatment	
Surgery	462 (88.5%)
Hormonal (Fertility sparing reasons)	21 (4.0%)
Hormonal (Not fit for surgery)	36 (6.9%)
Radiotherapy	3 (0.6%)
Adjuvant treatment	
Yes	234 (44.9%)
No	287 (55.1%)
Recurrence	
Yes	76 (14.6%)
No	445 (85.4%)
Survival status at end of follow up	
Alive	414 (79.3%)
Cancer-specific mortality	74 (14.2%)
Non-cancer related mortality	34 (6.5%)
Total	522 (100%)

### 3.2 Kaplan-Meier survival estimation and Cox regression analysis

The median follow-up was 40 months (IQR 24–57 months). Overall survival rates were 95% (92–96%) at 12 months, 84% (81–87%) at 36 months and 76% (71–80%) at 60 months. Age, T2DM status, stage, histology, grade, LVSI and depth of myometrial invasion were all important predictors of overall survival. There was a 7% increased risk of death from any cause per unit increase in age (HR = 1.07, 95%CI 1.05–1.09, *p* < 0.001), however, there was no effect of BMI (HR = 0.99, 95%CI 0.98–1.01, *p* = 0.576). Compared to those without, women with T2DM had a 93% higher mortality risk (HR = 1.93, 95%CI 1.28–2.99, *p* = 0.002). Women with advanced disease (stage III/IV) had a three-fold higher risk of death (HR = 3.01, 95%CI 1.99–4.57, *p* < 0.001) than those with early-stage disease (stage I/II). Women with non-endometrioid tumors had a three-fold higher mortality risk (HR = 3.06, 95%CI 2.09–4.48, *p* < 0.001) than those with endometrioid tumors, and women with grade III disease had a near three-fold higher mortality risk (HR = 2.99, 95%CI 2.04–4.39, *p* < 0.001) than those with grade I/II disease. LVSI and deep myometrial invasion were also associated with higher risks of death (HR = 2.27, 95%CI 1.55–3.31, p < 0.001 and HR = 1.78, 95%CI 1.22–2.60, *p* = 0.003, respectively).

Of the 108 deaths, 74(68.5%) were due to endometrial cancer. Cancer-specific survival rates were 96% (94–97%) at 12 months, 89% (85–91%) at 36 months and 82% (77–86%) at 60 months. Age at diagnosis (HR = 1.06, 95%CI 1.03–1.08, *p* < 0.001), T2DM status (HR = 1.68, 95%CI 1.01–2.78, *p* = 0.040), stage (HR = 4.97, 95%CI 3.11–7.93, p < 0.001), grade (HR = 5.86, 95%CI 3.51–9.80, p < 0.001), histology (HR = 5.14, 95%CI 3.20–8.23, p < 0.001), LVSI (HR = 3.51 95%CI 2.21–5.57, p < 0.001) and deep myometrial invasion (HR = 2.24, 95%CI 1.42–3.55, *p* = 0.01) were important predictors of cancer-specific survival in univariable analyses.

Overall, there were 76 recurrences with a median time to recurrence of 14 months (range 1–54 months). The recurrence-free survival estimates for the whole cohort were 93% (90–95%) at 12 months, 83% (79–86%) at 36 months and 78% (75–84%) at 60 months. Age at diagnosis (HR = 1.04, 95%CI 1.02–1.07, *p* < 0.001), stage (HR = 4.63, 95%CI 2.91–7.37, p < 0.001), grade (HR = 4.50, 95%CI 2.80–7.23, p < 0.001), histology (HR = 3.62, 95%CI 2.31–5.69, p < 0.001), LVSI (HR = 4.14, 95%CI 2.62–6.54, p < 0.001), deep myometrial invasion (HR = 2.29, 95%CI 1.46–3.60, p < 0.001) and T2DM status (HR = 1.75, 95%CI 1.07–2.88, *p* = 0.027) were important predictors of recurrence-free survival.

### 3.3 Pre-treatment CRP and endometrial cancer overall, cancer-specific and recurrence-free survival

**Fig. 1:**
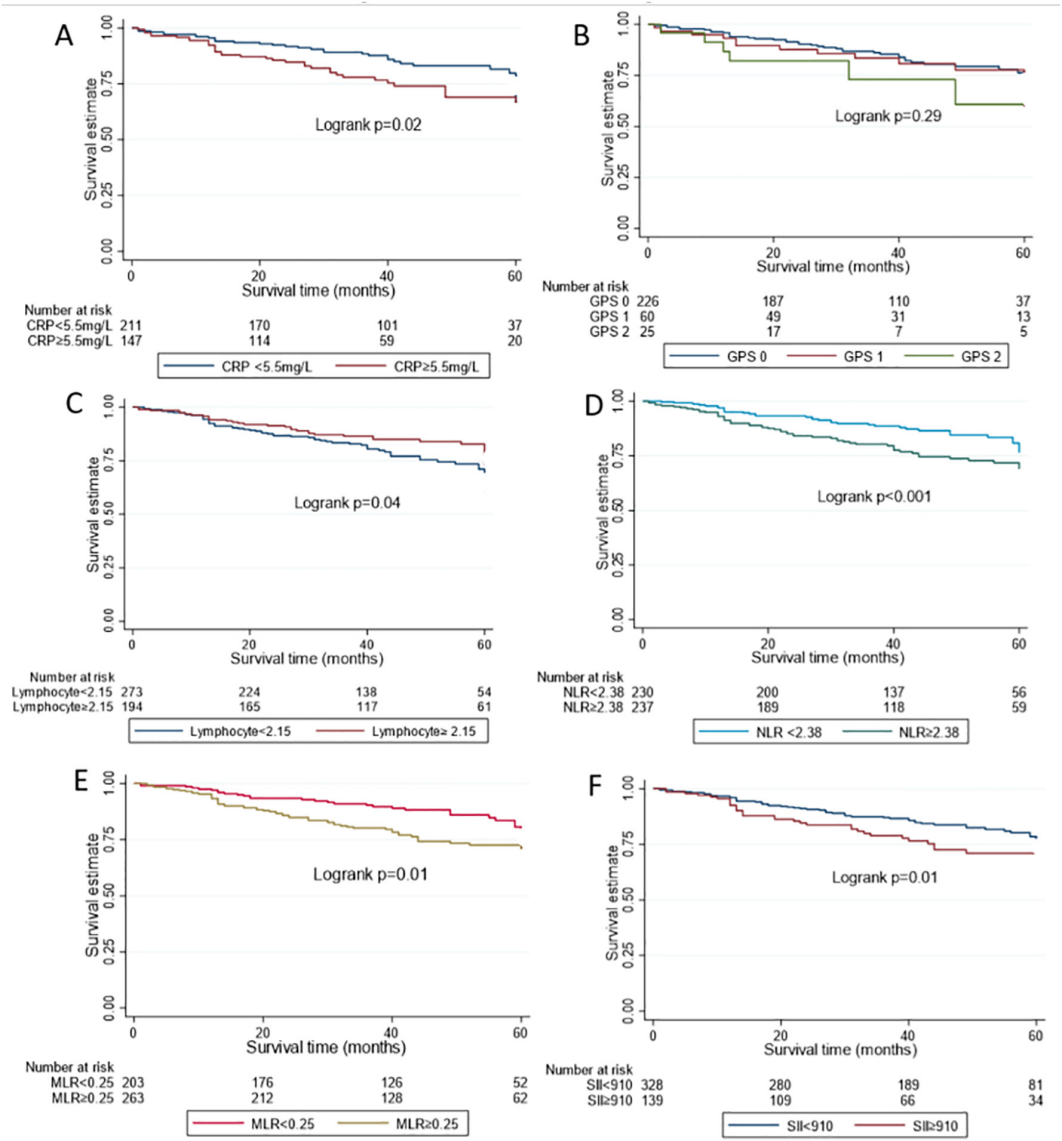
Kaplan-Meier survival curves showing survival estimates by CRP (A), GPS (B), Lymphocyte count (C), NLR (D), MLR (E) and SII (F) categories.

CRP values ranged from 0.2 mg/L to 158 mg/L with a median CRP of 4 mg/L and IQR of 1.7-10 mg/L. The optimal prognostic cut-off value for survival based on the ROC curve decision threshold analysis was 5.5 mg/L (specificity 61%, sensitivity 49%, AUC 0.55). A total of 147 women (41.1%) with pre-treatment CRP ≥ 5.5 mg/L were classed as having ‘high’ CRP whilst the remaining 211 with CRP < 5.5 mg/L were classed as having ‘low’ CRP. There was an association between CRP and BMI (Spearman's correlation coefficient 0.35, *p* < 0.001), T2DM status and primary treatment received, but no evidence for an association with age, socioeconomic status, stage, histology, grade, LVSI or myometrial invasion at the decision threshold of 5.5 mg/L (Table 2). At a higher threshold of 10 mg/L (used in the computation of GPS), there was evidence of an association between CRP and histology (*p* = 0.01) and tumor grade (*p* = 0.02). Women with high CRP had significantly higher overall and cancer-specific mortality rates than those with low CRP in both univariable and multivariable analyses (Fig. 1A, Table 3). There was no evidence of an effect of pre-treatment CRP on recurrence-free survival. When CRP was analysed as a continuous variable, the adjusted hazard ratios were 1.01(95%CI 1.00–1.03, *p* = 0.144, 1.02(95%CI 1.00–1.04, *p* = 0.057) and 1.00(95%CI 0.99–1.03, *p* = 0.860) for overall, cancer-specific and recurrence free survival respectively.

**Table 2:** Baseline socio-demographic characteristics stratified by CRP-based categories.

		Frequency	CRP <5.5 mg/L (Low)	CRP ≥5.5 mg/L (High)	p value	Frequency	GPS = 0	GPS=I/II	p value
Age (years)	<65	166	95(57.2%)	71(42.8%)	0.541	134	98(73.1%)	36(26.9%)	0.873
	≥65	192	116(60.4%)	76(39.6%)		177	128(72.3%)	49(27.7%)	
BMI (kg/m^2^)	Underweight	4	3(75.0%)	1(25.0%)	**0.001**	4	2(50.0%)	2(50.0%)	0.123
	Normal	52	34(65.4%)	18(34.6%)		51	35(68.6%)	16(31.4%)	
	Overweight	87	65(74.7%)	22(25.3%)		81	66(81.5%)	15(18.5%)	
	Obese	215	109(50.7%)	106(49.3%)		175	123(70.3%)	52(29.7%)	
FIGO stage	I	272	164(60.3%)	108(39.7%)	0.582	231	172(74.5%)	59(25.5%)	0.243
	II	37	18(48.7%)	19(51.4%)		33	20(66.6%)	13(39.4%)	
	III	44	26(59.1%)	18(40.9%)		42	31(73.8%)	11(26.2%)	
	IV	4	2(50.0%)	2(50.0%)		4	2(50.0%)	2(50.0%)	
Histology	Endometrioid	277	168(60.7%)	109(39.4%)	0.224	234	177(75.6%)	57(24.4%)	**0.040**
	Others	81	43(53.1%)	38(46.9%)		77	49(63.6%)	28(36.4%)	
Grade	I	169	96(56.8%)	73(43.2%)	0.104	132	93(70.5%)	39(29.5%)	**0.015**
	II	86	59(68.6%)	27(31.4%)		79	67(84.8%)	12(15.2%)	
	III	103	56(54.4%)	47(45.6%)		100	66(66.0%)	34(34.0%)	
LVSI	No	253	147(58.1%)	106(41.9%)	0.716	210	153(72.9%)	57(27.1%)	0.874
	Yes	103	62(60.2%)	41(39.8%)		100	72(72.0%)	28(28.0%)	
Myometrial invasion	<50%	231	136(58.9%)	95 (41.1%)	0.973	190	143(75.3%)	47(24.7%)	0.198
	≥50%	127	75(59.1%)	52(40.9%)		121	83(68.6%)	38(31.4%)	
Type 2 diabetes mellitus	No	285	179(62.8%)	106(37.2%)	**0.005**	252	188(74.6%)	64(25.4%)	0.117
	Yes	70	31(44.3%)	39(55.7%)		56	36(64.3%)	20(35.7%)	
Social quintile	I	135	75(55.6%)	60 (44.4%)	0.681	117	80(68.4%)	37 (31.6%)	0.268
	II	80	46(57.5%)	34(42.5%)		67	48(71.6%)	19(28.4%)	
	III	39	24(61.5%)	15(38.5%)		34	24(70.6%)	10(29.4%)	
	IV	60	40(66.7%)	20(33.3%)		53	45(84.9%)	8(15.1%)	
	V	44	26(59.1%)	18(40.9%)		40	29(72.5%)	11(27.5%)	
Primary treatment	Surgery	299	187(62.5%)	112(37.5%)	**0.004**	294	219(74.5%)	75(25.5%)	**0.003**
	Hormonal	57	23(40.4%)	34 (59.6%)		17	7(41.2%)	10(58.8%)	
	Radiotherapy	2	1(50.0%)	1(50.0%)		–	–	–	
Adjuvant therapy	No	205	116(56.6%)	89(43.4%)	0.261	165	123(74.5%)	42(25.5%)	0.408
	Yes	152	95(62.5%)	57(37.5%)		145	102(70.3%)	43(29.7%)	
Recurrence	No	316	190(60.1%)	126(39.9%)	0.275	272	197(72.4%)	75(27.6%)	0.800
	Yes	41	21(51.2%)	20(48.8%)		39	29(74.4%)	10(25.6%)	
Alive status	No	73	37(50.7%)	36(49.3%)	0.108	64	44(68.8%)	20(31.2%)	0.430
	Yes	285	174(61.1%)	111(38.9%)		247	182(73.7%)	65(26.3%)	

Bold indicates p < 0.05.

**Table 3:** Cox regression analyses of pre-treatment CRP and endometrial cancer survival outcomes with crude and adjusted hazard ratios and 95% confidence intervals.

CRP categories	Unadjusted HR (95%CI)	p-Value	Adjusted HR (95%CI)	p-Value
Overall mortality				
CRP <5.5 mg/L	1.00		1.00	
CRP ≥5.5 mg/L	1.75 (1.09–2.80)	0.020	1.68 (1.00–2.81)	0.049

Cancer-specific mortality				
CRP <5.5 mg/L	1.00		1.00	
CRP ≥5.5 mg/L	2.07 (1.13–3.76)	0.018	2.04 (1.03–4.02)	0.040

Disease recurrence				
CRP <5.5 mg/L	1.00		1.00	
CRP ≥5.5 mg/L	1.46 (0.79–2.69)	0.229	1.13 (0.58–2.20)	0.712

Adjusted model includes age, BMI, histology, grade, FIGO stage, LVSI, depth of myometrial invasion, T2DM status and treatment received.

### 3.4 GPS, mGPS and endometrial cancer overall, cancer-specific and recurrence-free survival

GPS was available for 311 women, including 226(72.7%) with GPS 0, 60(19.3%) with GPS 1 and 25(8.0%) with GPS 2. mGPS categories included 237 (76.2%) with mGPS 0, 49 (15.8%) with mGPS 1, and 25 (8.0%) with mGPS 2. There was a weak correlation between GPS and BMI (Spearmans rank correlation coefficient 0.12, *P* < 0.04). There was an association between GPS and histology, grade and treatment received (Table 2). Similarly, mGPS was associated with histology (*p* = 0.007), tumor grade (*p* = 0.010), and primary treatment (*p* = 0.003). There was no evidence of an effect of GPS or mGPS on overall, cancer-specific or recurrence-free survival (Fig. 1B, Table S1).

### 3.5 Lymphocyte count, Neutrophil-Lymphocyte Ratio, Monocyte-Lymphocyte Ratio and endometrial cancer overall, cancer-specific and recurrence-free survival

Lymphocyte counts were available for 467 women with values ranging from 0.4 × 10^9^/L to 6.62 × 10^9^/L, with a median value of 2.02 × 10^9^/L and IQR of 1.58–2.5 × 10^9^/L. The optimal prognostic cut-off for this parameter was 2.15 × 10^9^/L (specificity 58%, sensitivity 35%, AUC 0.47). Approximately 42% (194 women) had lymphocyte counts ≥2.15 × 10^9^/L. There was an association between lymphocyte count ≥2.15 × 10^9^/L and BMI but no evidence for an association with any other clinico-pathological variable (Table S2). Women with lymphocyte counts ≥2.15 × 10^9^/L had higher overall mortality rates than women with lymphocyte counts <2.15 × 10^9^/L on univariable analysis (HR = 0.65, 95%CI 0.43–0.99, *p* = 0.04) (Fig. 1C). There was no evidence of an effect of lymphocyte count on overall (adjusted HR = 0.67, 95%CI 0.42–1.04, *p* = 0.08), cancer-specific (adjusted HR = 0.68, 95%CI 0.39–1.16, *p* = 0.16) or recurrence-free survival (adjusted HR = 0.74, 95%CI 0.44–1.25, *p* = 0.27) at a prognostic threshold of 2.15 × 10^9^/L, in multivariable analyses.

NLR values ranged from 0.28 to 32 (median 2.39) and IQR 1.79–3.27. The optimal prognostic cut-off was 2.38 (specificity 53%, sensitivity 65%, AUC 0.59). Approximately 50% of women (*n* = 237) had an NLR ≥2.38. There was an association between NLR prognostic categories and age, stage, grade, LVSI and depth of myometrial invasion (Table S2). Women with NLR ≥2.38 had higher overall and cancer-specific mortality rates than women with NLR <2.38 (HR = 1.86, 95%CI 1.23–2.81, *p* = 0.003 and HR = 1.73, 95%CI 1.08–2.79, *p* = 0.020, respectively) in univariable analyses (Fig. 1D), however, after adjusting for confounding factors, there was no evidence of an effect of NLR on overall, cancer-specific or recurrence-free survival.

MLR values ranged from 0.06 to 0.80 (median 0.27) and IQR 0.21–0.35. The optimal prognostic threshold based on ROC analysis was 0.25 (specificity 49%, sensitivity 66%, AUC 0.58). A total of 263 women (56.4%) had MLR values ≥0.25. There was an association between MLR prognostic categories and age, BMI, histology, grade and depth of myometrial invasion (Table S2). Women with MLR ≥0.25 had higher overall mortality, cancer-specific mortality and recurrence rates than women with MLR <0.25 (HR = 1.66, 95%CI 1.09–2.50, *p* = 0.02; HR = 1.64, 95%CI 1.01–2.67, *p* = 0.04 and HR = 1.71, 95%CI 1.05–2.79, *p* = 0.03, respectively) in univariable analyses (Fig. 1E). However, after adjusting for confounding factors, there was no evidence of an effect of MLR on overall, cancer-specific or recurrence-free survival.

### 3.6 Systemic Immune Inflammation Index (SII) and endometrial cancer overall, cancer-specific and recurrence-free survival

SII data were available for 467 women and had values ranging from 53 to 9366 (median value 678, IQR 477–1009). The optimal SII cut-off value based on the ROC curve decision threshold was 910 (sensitivity 39%, specificity 72%, AUC 0.65). A total of 139 women (29.8%) had SII values ≥910. There was an association between SII values ≥910 and standard pathological prognostic factors, including stage, histology, grade, LVSI and depth of myometrial invasion (Table S2). Over the study period, 23 women with SII values ≥910 (16.5%) recurred compared to 50 women with values <910 (15.2%), *p* = 0.700. There was a significantly higher overall mortality rate in women with SII ≥910 compared to women with SII <910 (28.1% vs 18.6% respectively, *p* = 0.023). Women with pre-treatment SII values ≥910 had a higher rate of all-cause mortality than those with SII values <910 (HR = 1.58, 95%CI 1.05–2.36, *p* = 0.026) on univariable analysis (Fig. 1F). However, after adjusting for confounding factors, there was no evidence of an effect of SII on overall, cancer-specific or recurrence-free survival.

## 4 Discussion

### 4.1 Main findings

Here, we show evidence for the potential utility of CRP as a prognostic biomarker in endometrial cancer. Women with a high pre-treatment CRP at a decision threshold of 5.5 mg/L had a 68% increase in overall mortality and a two-fold higher cancer-specific mortality risk compared to those with low CRP. Absolute lymphocyte count, NLR, MLR and SII were associated with aggressive tumor parameters including stage, histology, grade, LVSI and deep myometrial invasion, but when these and clinical prognostic factors were controlled for, there was no evidence that lymphocyte-based scores are associated with overall, cancer-specific or recurrence-free survival.

### 4.2 Strengths and limitations

Our study benefits from a large cohort of endometrial cancer patients recruited to population-based studies with broad inclusion criteria, alleviating concerns about selection bias. The availability of high quality socio-demographic and clinico-pathological data allowed for robust correction for confounding factors and effect modifiers. Classically applied endometrial cancer prognostic parameters, including stage, grade, histological subtype, LVSI and depth of myometrial invasion, all demonstrated the expected associations. The lack of data on ethnicity, surgical approach and molecular subgroup is a limitation of our work that may lead to an over- or under-estimation of survival outcomes. The relatively small sample size for the CRP cohort reduces the precision of our estimates. We were not able to validate the utility of data-derived cut-offs either in a separate cohort or through cross validation within this cohort, due to low overall numbers. Thus further work is needed before CRP can be introduced as a prognostic biomarker in routine clinical practice. As a single center study of mostly White British women, we cannot necessarily extrapolate our study findings to women from other centers, nationalities or ethnic backgrounds.

### 4.3 Interpretation

Obesity plays a strong etiological role in endometrial carcinogenesis and is characterized by a chronic low-grade inflammatory state [32,33]. Inflammation predisposes to tumorigenesis by damaging DNA, stimulating angiogenesis and potentiating pro-proliferative and anti-apoptotic processes [14,34]. The inflammatory cytokines that drive these processes include IL-1, IL-6, tumor necrosis-alpha and interferon-gamma [12]. These stimulate the production of CRP, an acute-phase protein that is produced by the liver and released directly into the blood. The potential prognostic utility of CRP has been investigated in many solid malignancies [27], although endometrial cancer has been relatively understudied [16,18,28]. A multicenter study of 403 surgically treated patients found CRP was an independent prognostic marker for endometrioid endometrial cancer [16]. CRP was associated with stage, but not tumor grade, LVSI or age at diagnosis [16], and the study failed to control for other important prognostic parameters, for example BMI, T2DM and depth of myometrial invasion. A small study of 176 women with type 1 endometrial cancer found high pre-operative CRP levels were associated with increased all-cause mortality [28] but the authors failed to include non-endometrioid tumors in their analysis or adjust for important confounders. Another small study of just 110 women with endometrial cancer demonstrated an association between pre-treatment CRP levels and both overall and disease-free survival [18]. In addition, Saijo and colleagues reported GPS 2 to be an independent predictor of survival and recurrence in endometrial cancer [19]. Here, we show that CRP is a strong predictor of endometrial cancer survival outcomes following robust adjustment for important clinico-pathological confounders. CRP was associated with BMI and T2DM status, consistent with previous work [[35], [36], [37], [38]], and at a higher threshold of 10 mg/L, demonstrated an association with histological subtype and grade, but not stage, LVSI or depth of myometrial invasion. If validated in a larger cohort, these findings have important clinical implications. CRP is a simple, easy to perform, low-cost test that can aid the identification of women at a higher risk of death from endometrial cancer. High CRP was recorded for approximately 41% of patients, for whom bespoke management strategies and careful follow-up may be justified. When considered alongside standard clinico-pathological characteristics, CRP may help risk stratify patients and guide decisions about adjuvant therapy, however, there is currently no evidence to support its role as a biomarker for disease recurrence. An endometrial cancer blood test has strong appeal for patients and clinicians alike. “Are blood tests useful in predicting survivorship and/or recurrent disease?” ranked 5th most important endometrial cancer research priority in the James Lind Alliance priority setting partnership [39] of patients, carers, healthcare professionals and members of the general public.

SII is a novel composite indicator of inflammation and a promising prognostic biomarker for several solid adult malignancies [40]. A meta-analysis of 2724 patients showed that elevated SII was associated with poor overall survival and increased risk of lymph node metastasis in patients with gynecological malignancies [29]. An association between SII and disease-free survival was noted in women with ovarian and breast cancer but no endometrial cancer studies were included in the review. Subsequent studies include a retrospective analysis of 442 patients of Japanese descent with a mean BMI of 23 kg/m^2^, which found SII was an independent prognostic factor in endometrial cancer. These findings cannot necessarily be extrapolated to non-Japanese endometrial cancer patients with elevated BMI [29]. Further data come from a study of 155 women with FIGO stage I-III endometrial cancer treated with postoperative external beam radiotherapy, which found elevated SII was associated with decreased overall survival [24]. Whilst the authors attempted adjustment for confounding variables, histological subtype and tumor grade did not correlate with survival outcomes, raising concerns about the statistical power of the study. Another study of 101 women with endometrial cancer reported a higher SII was associated with shorter progression-free and overall survival times, but was limited by small numbers [30]. To our knowledge, ours is the largest study to date to investigate the prognostic relevance of SII in women with endometrial cancer. We showed that SII is linked to aggressive endometrial cancer phenotypic parameters, specifically FIGO stage, histology, LVSI and deep myometrial invasion, but when these are adjusted for, there is no evidence for an effect of SII on survival outcomes at these prognostic thresholds. Whilst there are no clinically validated prognostic thresholds for markers like SII, and since applied thresholds vary between studies, a different conclusion might be reached based on alternative thresholds. Well-designed studies with adequate sample sizes are now needed to confirm the true value of SII as a prognostic biomarker in endometrial cancer and to identify optimal decision thresholds.

We and others have shown that absolute lymphocyte count, NLR and MLR are associated with adverse clinico-pathologic factors in endometrial cancer [30,[41], [42], [43]]. A retrospective analysis of 197 endometrial cancer patients investigated the potential utility of NLR to predict lymph node metastasis [41] while another study found a NLR >2.41 predicted cervical stromal involvement in endometrioid endometrial cancer [42]. These findings are consistent with those of a meta-analysis by Pergialiotis and colleagues, who showed NLR was associated with advanced-stage disease, positive lymph nodes, LVSI and distant metastases [43]. A retrospective series of 605 surgically treated endometrial cancer patients found NLR, but not MLR, was an independent prognostic factor. NLR was linked to advanced stage while MLR was associated with advancing age [21]. This study controlled for age, stage, histology and LVSI and is thus open to residual confounding by BMI, T2DM status, depth of myometrial invasion amongst others. A retrospective review of 510 surgically managed endometrial cancer patients of Chinese descent found NLR to be an independent prognostic marker [44]. Several studies support these findings [20,22,[45], [46], [47]], while others do not [17,41,48]. A systematic review of nine studies and 3390 patients concluded that elevated NLR has potential as a prognostic marker in women with endometrial cancer [49], but was limited by, marked heterogeneity of included studies with respect to NLR thresholds and small study sizes.

## 5 Conclusion

In this study, we found pre-treatment CRP to be an independent prognostic biomarker in endometrial cancer. Women with a high CRP at a decision threshold of 5.5 mg/L had a two-fold increased risk of death compared to women with low CRP. If validated in an independent cohort, CRP could provide a simple, low-cost prognostic test that has the potential to refine pre-treatment risk assessment and guide decisions about adjuvant treatment in endometrial cancer.

## Contribution to authorship

Supervision and funding acquisition, EJC; Conceptualization, KN and EJC; study design, KN and EJC; data extraction, YLW, CEB, NCR, KN, EJC; statistical analysis, KN; writing, original draft preparation, KN, EJC; all authors provided critical comments, read, and approved the final version for publication.

## Details of ethics approval

This study uses data from a prospectively maintained database of women with endometrial cancer, participating in clinical research at St Mary's Hospital, Manchester. All women gave written, informed consent for their pseudo-anonymized clinical data to be used in future research. The primary research studies were: Metformin (North West Research Ethics Committee, NW REC, 11/NW/0442, approved 19 August 2011), Weight loss (NW REC, 12/NW/0050, approved 23 January 2012), PREMIUM (NW REC, 14/NW/1236, approved 23 September 2014), PETALS (NRES Committee North West, Lancaster, 15/NW/0733, approved 18 September 2015) and DETECT (NW REC, Greater Manchester, 16/NW/0660, approved 16 September 2016).

## Funding

KN is supported by a 10.13039/501100000289Cancer Research UK (CRUK) Manchester Cancer Research Centre Clinical Research Fellowship (C147/A25254) and the 10.13039/100010269Wellcome Trust Manchester Translational Informatics Training Scheme. NCR is supported by a 10.13039/501100000272National Institute for Health Research (NIHR) Academic Foundation Program. YLW is supported by a NIHR Academic Clinical Lectureship. CEB is supported by a Manchester University NHS Foundation Trust Clinical Research Fellowship. EJC is supported by the 10.13039/100014653NIHR Manchester Biomedical Research Centre (IS-BRC-1215-20007) and an NIHR Advanced Fellowship (NIHR300650).

## Declaration of Competing Interest

None declared.

## Appendix A Supplementary data

Supplementary data Image 1
