# Supplementary material for: Pre-treatment inflammatory parameters predict survival from endometrial cancer: A prospective database analysis
Source: Gynecol Oncol. 2022 Jan;164(1):146–53. doi: 10.1016/j.ygyno.2021.11.009 (PMC8802781; doi:10.1016/j.ygyno.2021.11.009)
Supplement: Supplementary file 1 — Supplementary data [file mmc1.docx]

**Table S1: Cox regression analysis of Glasgow Prognostic Score (GPS)/modified GPS and endometrial cancer survival outcomes with crude and adjusted hazard ratios and 95% confidence intervals.**

| **Categories** | **Unadjusted HR(95% CI)** | **p-value** | **Adjusted HR(95%CI)** | **p-value** |
| --- | --- | --- | --- | --- |
| **Overall Mortality** | | | | |
| GPS/mGPS 0 | 1.00 |  | 1.00 |  |
| GPS 1 or 2 | 1.29 (0.76-2.20) | 0.351 | 1.22 (0.68-2.20) | 0.510 |
| mGPS 1 or 2 | 1.47(0.85-2.55) | 0.170 | 1.34 (0.73-2.46) | 0.350 |
| **Cancer-Specific Mortality** | | | | |
| GPS/mGPS 0 | 1.00 |  | 1.00 |  |
| GPS 1 or 2 | 1.10 (0.56-2.15) | 0.788 | 1.01 (0.47-2.12) | 0.990 |
| mGPS 1 or 2 | 1.44 (0.74-2.82) | 0.287 | 1.22 (0.58-2.60) | 0.601 |
| **Disease Recurrence** | | | | |
| GPS/mGPS 0 | 1.00 |  | 1.00 |  |
| GPS 1 or 2 | 0.96 (0.47-1.97) | 0.914 | 0.75 (0.35-1.60) | 0.454 |
| mGPS 1 or 2 | 1.21 (0.59-2.48) | 0.607 | 0.94 (0.44-2.01) | 0.880 |

Adjusted model includes age, BMI, histology, grade, FIGO stage, LVSI, depth of myometrial invasion, T2DM status and treatment received.

**Table S2:** **Baseline socio-demographic characteristics stratified by lymphocyte-based categories**

|  |  | Frequency | Lymphocyte <2.15 | Lymphocyte ≥2.15 | p  value | NLR<2.38 | NLR≥2.38 | p value | MLR<0.25 | MLR≥0.25 | p value | SII<910 | SII≥910 | p  value |
| --- | --- | --- | --- | --- | --- | --- | --- | --- | --- | --- | --- | --- | --- | --- |
| Age (years) | <65 | 200 | 107(53.5%) | 93(46.5%) | 0.060 | 113(56.5%) | 87(43.5%) | **0.007** | 109(54.5%) | 91(45.5%) | **<0.001** | 143(71.5%) | 57(28.5%) | 0.605 |
|  | ≥65 | 267 | 166(62.2%) | 101(37.8%) |  | 117(43.8%) | 150(56.2%) |  | 94(35.3%) | 172(64.7%) |  | 185(69.3%) | 82(30.7%) |  |
| BMI (kg/m^2^) | Underweight | 5 | 4(80.0%) | 1(20.0%) | **<0.001** | 0(0.0%) | 5(100%) | 0.052 | 0(0.0%) | 5(100%) | **0.023** | 1(20.0%) | 4(80.0%) | 0.119 |
|  | Normal | 80 | 63(78.8%) | 17(21.2%) |  | 34(42.5%) | 46(57.5%) |  | 26(32.5%) | 54(67.5%) |  | 55(68.7%) | 25(31.3%) |  |
|  | Overweight | 122 | 69(56.6%) | 53(43.4%) |  | 59(48.4%) | 63(51.6%) |  | 55(45.5%) | 66(54.5%) |  | 86(70.5%) | 36(29.5%) |  |
|  | Obese | 260 | 137(52.7%) | 123(47.3%) |  | 137(52.7%) | 123(47.3%) |  | 122(46.9%) | 138(53.1%) |  | 186(71.5%) | 74(28.5%) |  |
| FIGO stage | I | 342 | 201(58.8%) | 141(41.2%) | 0.455 | 184(53.8%) | 158(46.2%) | **0.004** | 158(46.3%) | 183(53.7%) | 0.098 | 251(73.4%) | 91(26.6%) | **0.024** |
|  | II | 51 | 25(49.0%) | 26(51.0%) |  | 22(43.1%) | 29(56.9%) |  | 21(41.2%) | 30(58.8%) |  | 35(68.6%) | 16(31.3%) |  |
|  | III | 66 | 41(62.1%) | 25(37.9%) |  | 21(31.8%) | 45(68.2%) |  | 22(33.3%) | 44(66.7%) |  | 38(57.6%) | 28(42.4%) |  |
|  | IV | 7 | 5(71.4%) | 2(28.6%) |  | 2(28.6%) | 5(71.4%) |  | 1(14.3%) | 6(85.7%) |  | 3(42.9%) | 4(57.1%) |  |
| Histology | Endometrioid | 339 | 196(57.8%) | 143(42.2%) | 0.647 | 175(51.6%) | 164(48.4%) | 0.095 | 159(46.9%) | 180(53.1%) | **0.017** | 248(73.2%) | 91(26.8%) | **0.025** |
|  | Others | 128 | 77(60.2%) | 51(39.8%) |  | 55(43.0%) | 73(57.0%) |  | 44(34.7%) | 83(65.4%) |  | 80(62.5%) | 48(37.5%) |  |
| Grade | I | 191 | 106(55.5%) | 85(44.5%) | 0.309 | 111(58.1%) | 80(41.9%) | **0.006** | 99(51.8%) | 92(48.2%) | **0.008** | 140(73.3%) | 51(26.7%) | 0.065 |
|  | II | 108 | 61(56.5%) | 47(43.5%) |  | 48(44.4%) | 60(55.6%) |  | 44(40.7%) | 64(59.3%) |  | 81(75.0%) | 27(25.0%) |  |
|  | III | 168 | 106(63.1%) | 62(36.9%) |  | 71(42.3%) | 97(57.7%) |  | 60(35.9%) | 107(64.1%) |  | 107(63.7%) | 61(36.3%) |  |
| LVSI | No | 321 | 188(58.6%) | 133(41.4%) | 0.972 | 174(54.2%) | 147(45.8%) | **0.001** | 146(45.6%) | 174(54.4%) | 0.115 | 234(72.9%) | 87(27.1%) | **0.044** |
|  | Yes | 143 | 84(58.7%) | 59(41.3%) |  | 53(37.1%) | 90(62.9%) |  | 54(37.8%) | 89(62.2%) |  | 91(63.6%) | 52(36.4%) |  |
| Myometrial invasion | <50% | 293 | 163(55.6%) | 130(44.4%) | 0.108 | 162(55.3%) | 131(44.7%) | **0.001** | 144(49.3%) | 148(50.7%) | **0.001** | 219(74.7%) | 74(25.3%) | **0.006** |
|  | ≥50% | 174 | 110(63.2%) | 64(36.8%) |  | 68(39.1%) | 106(60.9%) |  | 59(33.9%) | 115(66.1%) |  | 109(62.6%) | 65(37.4%) |  |
| History of T2DM | No | 377 | 229(60.7%) | 148(39.3%) | 0.053 | 186(49.3%) | 191(50.7%) | 0.710 | 170(45.2%) | 206(54.8%) | 0.153 | 260(69.0%) | 117(31.0%) | 0.291 |
|  | Yes | 87 | 43(49.4%) | 44(50.6%) |  | 41(47.1%) | 46(52.9%) |  | 32(36.8%) | 55(63.2%) |  | 65(74.7%) | 22(25.3%) |  |
| Social quintile | I | 173 | 93(53.8%) | 80(46.2%) | 0.453 | 81(46.8%) | 92(53.2%) | 0.792 | 73(42.2%) | 100(57.8%) | 0.1333 | 122(70.5%) | 51(29.5%) | 0.954 |
|  | II | 111 | 65(58.6%) | 46(41.4%) |  | 56(50.5%) | 55(49.5%) |  | 57(51.4%) | 54(48.6%) |  | 76(68.5%) | 35(31.5%) |  |
|  | III | 51 | 30(58.8%) | 21(41.2%) |  | 23(45.1%) | 28(54.9%) |  | 15(29.4%) | 36(70.6%) |  | 38(74.5%) | 13(25.5%) |  |
|  | IV | 81 | 51(63.0%) | 30(37.0%) |  | 42(51.9%) | 39(48.2%) |  | 36(44.4%) | 45(55.6%) |  | 57(70.4%) | 24(29.6%) |  |
|  | V | 51 | 34(66.7%) | 17(33.3%) |  | 28(54.9%) | 23(45.1%) |  | 22(44.0%) | 28(56.0%) |  | 35(68.6%) | 16(31.4%) |  |
| Primary Treatment | Surgery | 454 | 266(58.6%) | 188(41.4%) | 0.887 | 223(49.1%) | 231(50.9%) | 0.884 | 197(43.5%) | 256(56.5%) | 0.975 | 320(70.5%) | 134(29.5%) | 0.554 |
|  | Hormonal | 11 | 6(54.6%) | 5(45.4%) |  | 6(54.6%) | 5(45.4%) |  | 5(45.4%) | 6(54.6%) |  | 7(63.6%) | 4(36.4%) |  |
|  | Radiotherapy | 2 | 1(50.0%) | 1(50.0%) |  | 1(50.0%) | 1(50.0%) |  | 1(50.0%) | 1(50.0%) |  | 1(50.0%) | 1(50.0%) |  |
| Adjuvant therapy | No | 239 | 143(59.8%) | 96(40.2%) | 0.574 | 135(56.5%) | 104(43.5%) | **0.001** | 111(46.6%) | 127(53.4%) | 0.184 | 176(73.6%) | 63(26.4%) | 0.093 |
|  | Yes | 227 | 130(57.3%) | 97(42.7%) |  | 94(41.4%) | 133(58.6%) |  | 92(40.5%) | 135(59.5%) |  | 151(66.5%) | 76(33.5%) |  |
| Recurrence | No | 393 | 226(57.5%) | 167(42.5%) | 0.381 | 199(50.6%) | 194(49.4%) | 0.200 | 179(45.6%) | 214(54.5%) | 0.055 | 278(70.7%) | 115(29.4%) | 0.700 |
|  | Yes | 73 | 46(63.0%) | 27(37.0%) |  | 31(42.5%) | 42(57.5%) |  | 24(33.3%) | 48(66.7%) |  | 50(68.5%) | 23(31.5%) |  |
| Alive status | No | 100 | 65(65.0%) | 35(35.0%) | 0.134 | 35(35.0%) | 65(65.0%) | **0.001** | 34(34.0%) | 66(66.0%) | **0.030** | 61(61.0%) | 39(39.0%) | **0.023** |
|  | Yes | 367 | 208(56.7%) | 159(43.3%) |  | 195(53.1%) | 172(46.9%) |  | 169(46.2%) | 197(53.8%) |  | 267(72.8%) | 100(27.3%) |  |
